# Hepatic drug metabolism by CYP2D6 in critically ill adults with AKI: effect of phenotype and AKI severity

**DOI:** 10.1186/2197-425X-3-S1-A839

**Published:** 2015-10-01

**Authors:** K Lane, JJ Dixon, T Lee, A Johnston, R van Schaik, M van Fessem, IAM MacPhee, BJ Philips

**Affiliations:** St George's University Hospitals NHS Foundation Trust, Critical Care, London, United Kingdom; St George's University of London, Critical Care, London, United Kingdom; Analytical Services International Ltd, London, United Kingdom; Erasmus MC University Medical Center, Rotterdam, Netherlands; St George's University of London, Renal Medicine, London, United Kingdom

## Introduction

Hepatic drug metabolism by cytochrome P450 type 3A (CYP3A) is impaired in patients with acute kidney injury (AKI) [1]. The mechanisms underlying this are unclear. CYP2D6 is another clinically important hepatic CYP subtype, responsible for approximately 25% drug metabolism. Common phenotypic variation exists, the clinical relevance of which has not been previously demonstrated in the critically ill. Tramadol is a drug probe principally of CYP2D6 metabolism. M1 is formed by CYP2D6, and M2 by CYP3A4. In a pilot study, we showed that single time point determination of tramadol concentration 4 hours after IV tramadol bolus accurately predicts integral tramadol exposure and hence tramadol metabolism in critically ill adults [2].

## Objectives

We aimed to investigate if an association exists between CYP2D6 drug metabolism and AKI in critically ill adults. We also investigated the importance of genetically predicted CYP2D6 phenotype (poor, intermediate and extensive metabolisers) for drug metabolism in critically ill adults.

## Methods

As part of a study of drug metabolism, 72 consented critically ill adults received IV 10mg tramadol bolus at t= 0. Serum was collected prior to injection and at 4 hours for determination of serum tramadol and tramadol metabolite concentration by HPLC/MS/MS. Blood was analysed for CYP2D6 genotype. Data were collected to determine AKI severity by KDIGO classification. Glomerular filtration rate was assessed by simultaneous 4 hour urinary creatinine clearance (4CrCl) measurements. The clinical study was approved by the local ethics committee.

## Results

Baseline characteristics did not vary between groups

The median serum tramadol concentration at 4 hours for KDIGO 0-3 were 31.4, 29.6, 27.3 and 33.4 respectively. No significant difference in tramadol concentration by KDIGO class (Kruskal Wallis, p = 0.78), AKI duration, serum creatinine or urea or 4CrCl was found.

Tramadol concentration and those of its metabolites varied significantly with CYP2D6 phenotype (Kruskal Wallis, p = 0.005 for Tramadol, p = 0.0002 for M1, p = 0.0008 for M2)

When CYP2D6 phenotypes (IM, EM) were considered individually, no effect of AKI was seen on tramadol concentration (p = 0.83 and 0.50, Kruskal-Wallis).

## Conclusions

In contrast to CYP3A4, CYP2D6 metabolism does not appear to be affected by AKI severity or duration. Tramadol metabolism is strongly influenced by genetically predicted CYP2D6 phenotype in the critically ill and is likely to reflect wide variation in CYP2D6 substrate metabolism in general.

**Table 1:** Patient Characteristics median [range]

	All Patients	KDIGO 0	KDIGO 1	KDIGO 2	KDIGO 3
Patient No.	72	14	14	16	28
Patient Age	72 [19-88]	75 [22-88]	72.5 [50-82]	72.5 [59-83]	72 [19-88]
Baseline Serum Creatinine (mmol/L)	86 [34-259]	84 [69-112]	94 [58-162]	90 [61-259]	75 [34-221]
Illness Severity Score SOFA/APACHE II	8 [0-16]/ 20 [5-34]	7 [0-11]/ 18 [5-31]	8 [3-16]/ 18 [9-24]	7.5 [4-14]/ 17.5 [13-25]	10 [3-15]/ 20 [13-34]
Reason for admission Medical/Elective surgery/Emergency surgery	46/11/14	7/2/2	10/2/2	8/6/2	21/1/6
BMI	26.42 [17.3-40.4]	25.1 [19.3-39.8]	25.3 [17.3-37.1]	26.4 [20.1-39.3]	28.5 [17.8-40.4]
CYP2D6 Phenotype PM/IM/IMEM/EM/UEM/Unknown	7/24/2/36/1/2	1/6/0/7/0/0	2/3/0/9/0/0	1/5/1/8/1/0	3/10/1/12/0/2
Serum Creatinine at t = 0 (mmol/L)	178 [62-642]	97 [62-126]	156[66-223]	187 [81-280]	281 [74-642]
Time with AKI (h) [KDIGO 0 excluded]	35.5 [6-192]	NA	24 [6-72]	37.5 [6-144]	36 [16-192]

**Figure 1:**
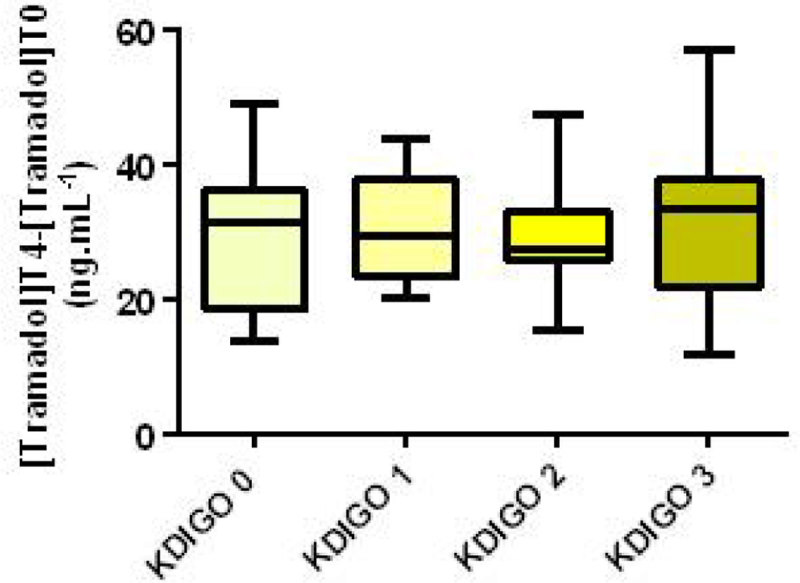
Figure 1

**Figure 2:**
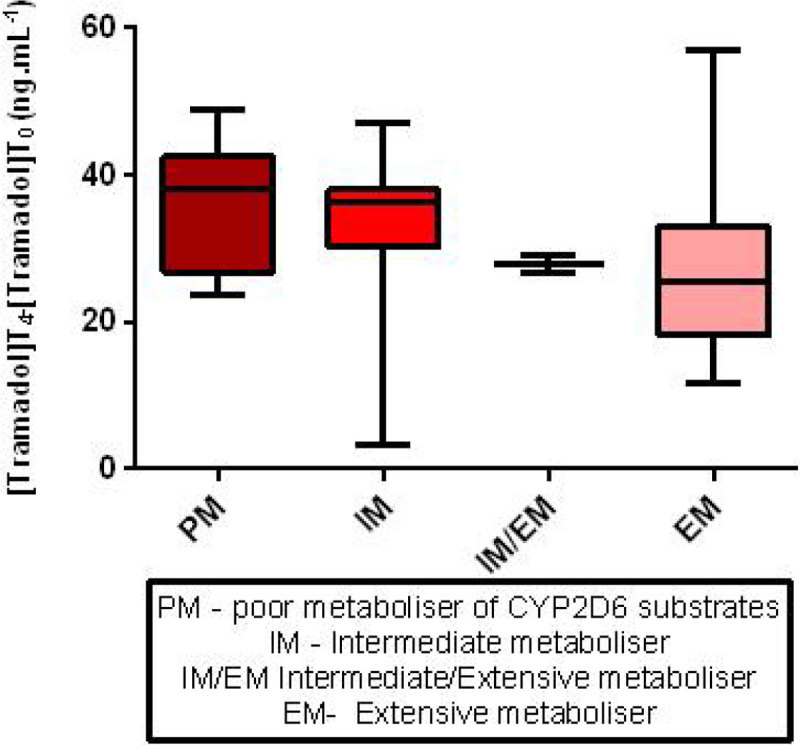
Figure 2

**Figure 3:**
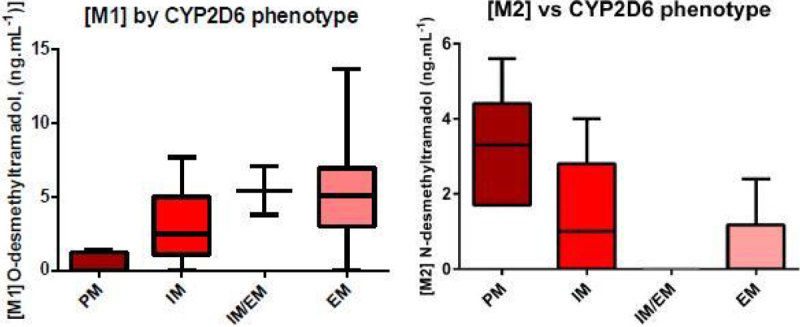
Figure 3

## Grant Acknowledgment

Dr Lane is supported by an ESICM Basic Sciences Award and St George´s Hospital Medical Charity Awards.
